# Use of Pleiotropy to Model Genetic Interactions in a Population

**DOI:** 10.1371/journal.pgen.1003010

**Published:** 2012-10-11

**Authors:** Gregory W. Carter, Michelle Hays, Amir Sherman, Timothy Galitski

**Affiliations:** 1 The Jackson Laboratory, Bar Harbor, Maine, United States of America; 2 Institute for Systems Biology, Seattle, Washington, United States of America; 3 EMD Millipore, Billerica, Massachusetts, United States of America; Princeton University, United States of America

## Abstract

Systems-level genetic studies in humans and model systems increasingly involve both high-resolution genotyping and multi-dimensional quantitative phenotyping. We present a novel method to infer and interpret genetic interactions that exploits the complementary information in multiple phenotypes. We applied this approach to a population of yeast strains with randomly assorted perturbations of five genes involved in mating. We quantified pheromone response at the molecular level and overall mating efficiency. These phenotypes were jointly analyzed to derive a network of genetic interactions that mapped mating-pathway relationships. To determine the distinct biological processes driving the phenotypic complementarity, we analyzed patterns of gene expression to find that the pheromone response phenotype is specific to cellular fusion, whereas mating efficiency was a combined measure of cellular fusion, cell cycle arrest, and modifications in cellular metabolism. We applied our novel method to global gene expression patterns to derive an expression-specific interaction network and demonstrate applicability to global transcript data. Our approach provides a basis for interpretation of genetic interactions and the generation of specific hypotheses from populations assayed for multiple phenotypes.

## Author Summary

Parallel advances in genotype and phenotype measurement technologies are yielding large-scale, multidimensional datasets that can potentially decipher the genetic etiology of complex traits. Understanding these data will require methods that combine the experimental power of molecular biology and the quantitative power of statistical genetics. In this work, we describe a novel approach that uses the complementary information encoded by multiple phenotypes in conjunction with genetic data to map genetic interaction networks in terms of quantitative variant-to-variant and variant-to-phenotype influences. We tested this method using a population of yeast strains with random combinations of five genetic mutations and derived an interaction network using molecular and colony-level assays of mating phenotypes. Distinct biological processes that underlie the two phenotypes were identified with gene expression analysis, validating the method's ability to exploit complementary biological information in multiple phenotypes. Our method generates data-driven models and testable hypotheses of how the genetic variation in a population combines to affect complex traits. It is designed to be flexible and scalable for application to populations with extensive genetic diversity.

## Introduction

Research in systems biology and genetics increasingly combines aspects of molecular biology with quantitative and statistical genetics. Genotyping and sequencing technologies allow large sample populations to be characterized at high or base-pair resolution. Parallel advances in quantitative phenotyping provide multidimensional descriptions of phenotypic states, often encompassing multiple molecular and physiological assays. Additionally, RNA transcript quantification is often used to provide a detailed view of cellular states. Translating this high-throughput, quantitative data into predictive models of health and disease will require new analytical methods to understand how genetic variants combine to influence multiple phenotypes.

One potentially powerful approach is the systematic study of genetic interactions. In molecular biology, genetic interaction analysis has been used to infer functional relationships such as activation, repression, and pathway ordering [1]. More recently, genome-scale interaction analysis has revealed functional genomic architecture in yeast [2]–[7], worm [8], [9], and fly [10], [11] model systems. Although equivalent genetic resources do not yet exist in mammalian model systems, new and forthcoming mouse populations will provide a basis for genetic interaction analysis in mammalian models [12]–[16]. However, the systematic interpretation of individual genetic interactions in terms of functional models has proven challenging in combinatorial genetic screens [5], [17], [18]. This ambiguity is even more pronounced in population-based studies, in which instances of statistical epistasis in highly powered studies rarely have clear biological interpretation. In some cases biological etiology can be resolved by pathway-based approaches that consider combinations of loci to detect polygenic risk [19], [20], but discovery is potentially limited by incomplete information on pathway structure and interactions. Studies of quantitative trait loci that affect gene expression (eQTL) identify multiple transcripts affected by interactions among genetic variants via *cis* and *trans* acting elements [21]–[27], providing insights into genetic influence on biological processes. Interaction studies in this approach can be limited by the cost of assaying sufficiently large sample numbers and ambiguous connections to physiological phenotypes. In all of these cases, methods that directly infer the structure of genetic networks would provide a data-driven model of how the genetic variation in a population organizes to affect complex traits.

**Figure 1:**
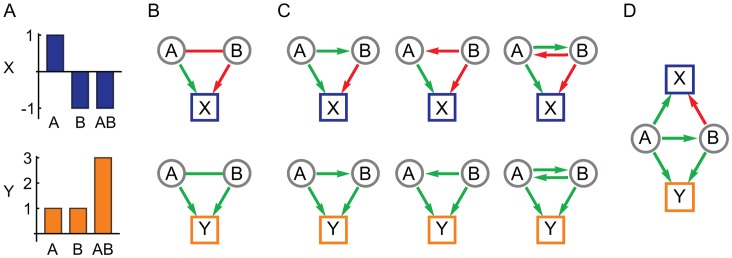
The use of pleiotropy to constrain the interpretation of epistasis. (A) The marginal effects of two genetic variants, A and B, in isolation and combination on two quantitative phenotypes, X and Y. (B) Variants A and B show negative (red) epistasis for X and positive (green) epistasis for Y, in addition to their individual effects in terms of positive (green) and negative (red) directional edges. (C) Possible model interpretations for the negative and positive epistasis for X and Y, respectively. (D) The simplest model of influences between A and B that is consistent with the observations of both X and Y.

In this paper we describe a new method to use complementary information in multiple phenotype measurements to infer the network structure of genetic interactions in terms of directional influences. The method requires two or more quantitative phenotypes that share some common genetic factors but are not completely correlated across all individuals. This reliance on partial pleiotropy is consistent with, and possibly a prerequisite to, the evolution of statistical epistasis [28]. It suggests a set of phenotypes that measure different aspects of the same complex trait or condition, such as a serum-based molecular biomarker paired with a whole-organism physiological measure. The method exploits the pleiotropy inherent in such a system to interpret instances of epistasis (in the statistical sense [29]) in terms of network models of genetic interactions that represent underlying biology [1]. The resulting network is constructed in terms of variant-to-variant influences, each quantifying how one gene variant affects the activity of a second variant, and variant-to-phenotype influences that quantify how each variant influences each phenotype. Interactions between variants can be either inter-locus (classical genetic interactions) or intra-locus (for a gene with multiple alleles). Our central assumption is that the spectrum of genetic interactions observed across multiple phenotypes is due to multiple manifestations of the same underlying network of variant-to-variant influences. By simultaneously analyzing interactions for multiple phenotypes, ambiguities are resolved to arrive at a consistent interpretation of each genetic interaction (Figure 1).

The method described here is designed for populations that harbor mixed genetic variation and therefore has conceptual and technical distinctions from methods designed for knock-out screens [2]–[4], [7]. First, since we are interested in modeling interactions for an arbitrarily large number of genetic variants in a population, the method is designed to assess individual variants rather than genes. The method therefore has the capability to address multiple allele forms at a given genetic locus, each with a potentially unique effect, and the inferred interactions describe the effect of each variant on every other variant's activity. Second, this work uses linear regression methods to estimate the single and double mutant effects across a population rather than by direct measurements that are only possible with engineered mutations on an isogenic strain background. Third, the extensive genetic diversity and large sample sizes of most intercross and natural populations require methods that can rapidly compute and assess the significance of interactions. Therefore the methods in this paper are mathematically simple and computationally fast. Finally, the networks derived in this work are constructed by analyzing each genetic interaction in isolation rather than obtaining a solution that simultaneously models all possible interactions [2]. Although a more comprehensive approach might be feasible with our test population, the abundance of genetic diversity and limited sample sizes of most populations will have insufficient statistical power for such analysis. Thus for computational and statistical tractability we develop methods to build networks from a very large number of pair-wise interactions, each inferred in isolation.

We tested our approach using a combination of molecular and colony-level assays of yeast mating competence across a population of strains with mutations of mating genes. We derived a genetic interaction network that is readily interpreted in terms of pathway genetics, mapping variant-to-variant interactions between mutations at different loci and variant-to-phenotype influences. We next analyzed gene expression to reveal the partially overlapping and partially distinct biological processes that underlie the phenotypic complementarity. We further analyzed the gene expression data to derive a similar interaction network and demonstrate the method's applicability to eQTL data. We conclude with a discussion of possible applications, extensions, and limitations of the approach.

## Results

We studied a population containing five mutations known to affect mating competence (Table 1). Deletions of *FUS3* [30] and *FAR1* [31] cause mating defects, whereas deletions of *BAR1* [32] and *MSG5* [33], [34] induce hypersensitivity to pheromone. The *STE11-4* allele is a missense mutation that constitutively activates the pheromone signaling response [35]. We constructed, genotyped, and analyzed 218 *MATa* strains, each containing an independent assortment of the five perturbations (Methods). This constituted a population of mixed positive and negative effect mutations that are known to exhibit genetic interactions [36], [37], and therefore provided an effective test population for our analysis methods.

**Table 1:** Genetic perturbations in this study.

*Gene*	*Description * [*58*]	*Chromosome Position*	*Allele*
*STE11*	Signal transducing MEK kinase involved in pheromone response	ChrXII849865–852018	*STE11-4*
*FAR1*	Cyclin-dependent kinase inhibitor that mediates cell cycle arrest in response to pheromone; also forms a complex that may specify the direction of polarized growth during mating	ChrX126324–123832	*far1Δ*
*FUS3*	Mitogen-activated serine/threonine protein kinase involved in mating	ChrII192454–193515	*fus3Δ*
*MSG5*	Dual-specificity protein phosphatase; adaptive response to pheromone; dephosphorylates Fus3	ChrXIV529943–531412	*msg5Δ*
*BAR1*	Aspartyl protease secreted into the periplasmic space of *MATa* cells, cleaves and inactivates alpha factor allowing cells to recover from alpha-factor-induced cell cycle arrest	ChrIX322340–324103	*bar1Δ*

We quantified overall mating efficiency (ME) and molecular pheromone response (PR) for each strain (Methods). The PR phenotype shares many genetic components with the ME phenotype and therefore ensured a degree of pleiotropy. However, because the PR assay was more narrowly focused on pheromone signaling, PR and ME were not expected to be entirely redundant. For example, strains with moderate defects in pheromone signaling may be mating competent (or hyper-competent) due to compensatory mutations that affect other biological processes. As expected, the ME and PR phenotypes were significantly correlated (Pearson *r* = 0.48, *p* = 3.3×10^−14^). To maximize complementarity between these phenotypes, we performed singular value decomposition (SVD) (Methods). The two resulting eigentrait vectors were orthogonal, normalized combinations of the sum and difference of ME and PR, respectively. We refer to these composite phenotypes as eigentraits 1 and 2 (ET1 and ET2).

Although all five perturbations have known roles in mating, some of them did not exhibit significant effects when considered individually. For each eigentrait we performed single-locus regression on each of our five genetic perturbations to identify significant variants used as covariates in pair-wise scans (Methods). Significance was defined as effect coefficients at least 3.55 estimated standard errors from zero (*p*<0.01; Methods). The *far1Δ*, *bar1Δ*, and *fus3Δ* perturbations were identified as covariates for ET1 and the *far1Δ* and *bar1Δ* perturbations were identified as covariates for ET2. These perturbations correspond to genes with very strong known effects (*BAR1*) or downstream signaling activators (*FAR1* and *FUS3*). Any significant effects of the *STE11-4* and *msg5Δ* perturbations were masked by the other factors in single-locus scans.

**Figure 2:**
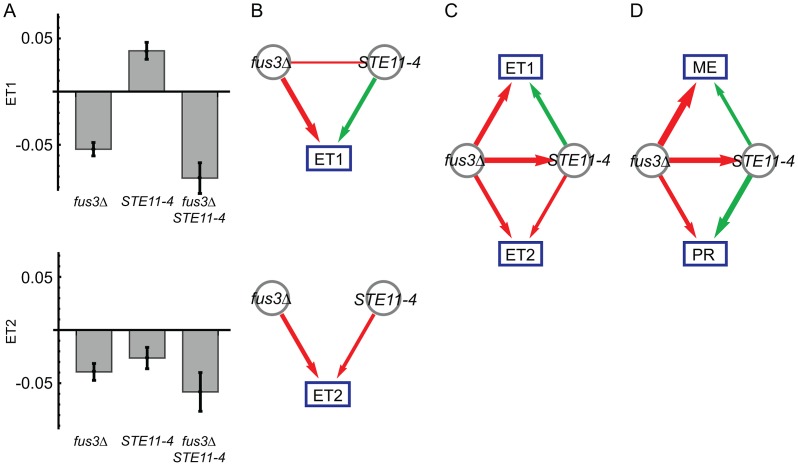
Genetic interactions between *fus3Δ* and *STE11-4* mutations, following **Figure 1**. (A) Modeled effects of *fus3Δ* and *STE11-4* mutations on eigentraits ET1 and ET2. (B) Significant coefficients and epistasis from pair-wise regression, in terms of positive (green) and negative (red) influences. Edge width represents interaction strength. (C) Interaction model consistent with both ET1 and ET2, in which *fus3Δ* suppresses *STE11-4*. (D) The same model expressed in terms of the original phenotypes of mating efficiency (ME) and pheromone response (PR).

**Figure 3:**
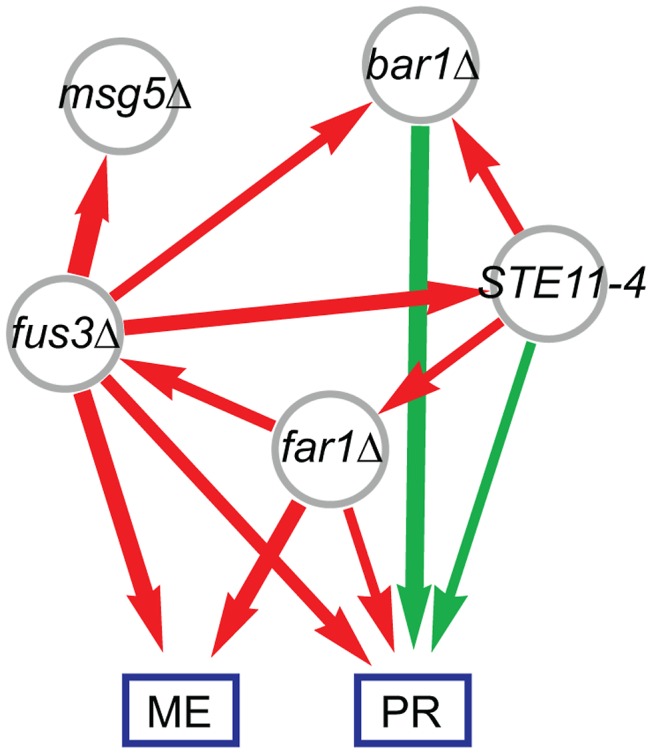
Combined genetic influence network for mating efficiency (ME) and pheromone response (PR) mapping positive (green) and negative (red) influences between mutations and on the phenotypes. Edge width represents interaction strength.

We next computed pair-wise models for each of the ten possible locus pairs to derive a genetic interaction network. One interaction, the inferred *fus3Δ* suppression of the *STE11-4* allele, is illustrated in Figure 2. We performed pair-wise linear regression on the eigentraits (Figure 2B; Methods, Eq. 3) and then reparametrized the interactions in terms of two variant-to-variant influences between perturbations (Methods, Eqs. 5 and 7). This procedure reinterprets statistical epistasis in terms of a genetic influences model. Figure 2C shows how the complementary information in our two eigentraits therefore determines that the *fus3Δ* perturbation reduces the effect of the *STE11-4* allele, ruling out alternative hypotheses such as a *STE11-4* enhancement of the *fus3Δ* effect that would be consistent with ET1 but not ET2. To determine the allelic effects on the ME and PR phenotypes, we recomposed the phenotype SVD for each pair-wise model and averaged over all models to obtain variant-to-phenotype influence coefficients (Figure 2D; Methods). This final step does not modify the inferred genetic interaction. Errors were estimated for all coefficients using standard least-squares regression and error propagation formulas for quantities computed from regression coefficients (Methods, Eq. 8). Our significance threshold was defined as *p*<0.05 based on genotype permutations and adjusted for multiple testing (Methods). All calculations were performed on a desktop PC using Mathematica software and each locus pair took approximately 30 milliseconds. We obtained a network of 12 significant interactions (Figure 3), including 6 variant-to-variant influences that fit the observed genetic interactions for both phenotypes. Results for all influence parameters are listed in Table S4.

**Figure 4:**
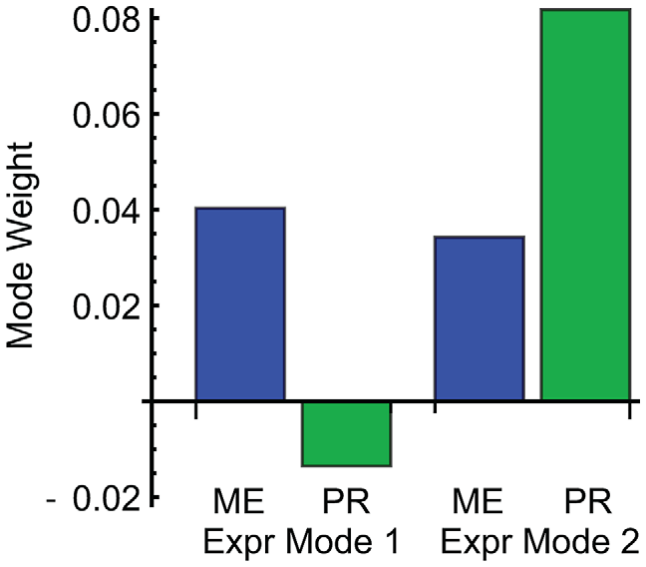
Weights of mating efficiency (ME) and pheromone response (PR) phenotypes for global expression patterns Mode 1 and Mode 2. ME is associated with both expression modes, while PR is primarily associated with Mode 2.

We combined ME and PR with gene expression data to identify complementary biological processes underlying the genetic interaction analysis. We selected a subset of strains representing the genetic diversity of the population and collected gene expression data after exposure to α-factor (Methods). To identify the global expression patterns we performed singular value decomposition (SVD) on the gene expression matrix (6208 genes across 92 strains) [38] (Methods). Because we sought to identify expression patterns that correspond to the ME and PR phenotypes, we included these quantitative phenotypes as additional rows in the gene expression matrix. The phenotypes were expressed relative to the unperturbed strain (wild-type) and normalized to a standard deviation of 2 in order to match the origin and scale of the gene expression data. Because these two rows were added to the expression patterns of thousands of genes, their contribution to the overall SVD patterns is negligible. The first two modes are the most dominant patterns in the data and account for 47% and 17% of the total variation in gene expression, respectively (Figure S3). By examining the ME and PR weight vectors for correspondence with each gene expression pattern, we found that Modes 1 and 2 capture the similarity and difference of these two phenotypes. The ME phenotype corresponds to both Modes 1 and 2, whereas the PR phenotype only corresponds to Mode 2 (Figure 4). Mode 3 further reinforces this conclusion. These different expression patterns therefore separate the biological processes shared between ME and PR (Expression Mode 2) and unique to ME (Expression Mode 1).

Gene set analysis revealed the biological functions of the genes associated with ME and PR. We identified sets of genes positively and negatively associated with each mode (Methods; Table S2). We queried each set of genes for enriched Gene Ontology (GO) annotations and transcription factor targets (Methods; Table 2 and Table S3). Expression Mode 1, the pattern associated with ME but not PR, was positively shown by a set of 169 genes that was most enriched in organic acid metabolic processes genes and transcriptional targets of Gcn4. The negative pattern, driven by 109 genes that were downregulated when mating efficiency is high, was shown by genes enriched in cell cycle and transcriptional targets of the SBF complex. Expression Mode 2, the pattern positively shown by 179 genes that were upregulated when both ME and PR are high, was enriched in mating genes and transcriptional targets of mating regulators. Many of the Mode 2 positive mating genes were also enriched in the Mode 1 positive gene set, but the Mode 1 negative metabolism and cell cycle genes were not present in Mode 2. This demonstrates that both ME and PR were reporting the changes in cellular morphology associated with mating. In contrast, the metabolic upregulation and cell-cycle downregulation were primarily associated with ME but not PR. We conclude that this difference constituted the complementary biological processes that allowed us to infer the genetic interaction network for mating efficiency and pheromone response (Figure 3).

**Table 2:** Summary of enriched functions and transcription factor binding targets for expression gene sets.

*Gene Set*	*GO Annotation*	*Number*	*p*	*Transcription Factor*	*Number*	*p*
Expr Mode 1, Positive, 169 genes	organic acid metabolic process	48	1.6×10^−20^	Gcn4	37	3.0×10^−19^
	cellular amino acid metabolic process	44	1.8×10^−20^	Ste12	35	3.5×10^−11^
	conjugation with cellular fusion	22	3.0×10^−13^			
Expr Mode 1, Negative, 109 genes	cell wall organization or biogenesis	19	2.3×10^−8^	Swi4	24	1.3×10^−14^
	cell division	22	6.0×10^−8^	Swi6	23	1.2×10^−13^
	cell cycle	32	8.3×10^−8^	Mbp1	21	2.0×10^−8^
Expr Mode 2, Positive, 179 genes	conjugation with cellular fusion	25	1.0×10^−15^	Ste12	67	1.7×10^−40^
	response to pheromone	18	4.4×10^−10^	Dig1	40	1.8×10^−24^
	karyogamy involved in conjugation with cellular fusion	8	9.4×10^−9^	Tec1	36	6.1×10^−15^

Full results are listed in Table S3.

The network model (Figure 3) was readily interpreted in terms how the perturbed genes affect the phenotypes and associated expression patterns. The expected *bar1Δ*, *fus3Δ*, *far1Δ*, and *STE11-4* single-perturbation effects on ME and/or PR were resolved. The strongest direct effects were from perturbations of canonical downstream pathway elements Far1 and Fus3. The *far1Δ* effect on ME was stronger than its effect on PR. Our gene expression analysis provides evidence that this was due to the role of Far1 in cell cycle regulation, a biological process that contributed to our ME phenotype but not PR (Results). The *bar1Δ* mutation had a very strong effect on PR but did not have a significant effect on ME. This was likely due to the fact that Bar1 degrades the alpha pheromone and thus its knockout leads to enhanced MAPK pathway activity [32].This Bar1 activity also enhances escape from pheromone-induced cell-cycle arrest to reinitiate proliferation [39].

The six variant-to-variant influences comprised a network view of how the perturbations affect one another and, in turn, the downstream phenotypes. The Msg5 phosphatase is known to affect mating by dephosphorylating the MAP kinase Fus3 [40], which is consistent with the suppression of *msg5Δ* effects by the *fus3Δ* allele. The *fus3Δ* allele also suppresses the effects of *bar1Δ* and *STE11-4*, two genes that are upstream of Fus3 in the canonical MAPK pathway. The moderate suppression of *far1Δ* by the *STE11-4* allele was consistent with the previous finding that MAPK pathway activation can provide compensatory cell cycle arrest in the absence of Far1 [37].

Our gene expression data set provided additional data for genetic interaction modeling. SVD modes have been demonstrated to be a suitable basis for statistical genetic analysis of gene expression [41] and we extended this concept to our pair-scan method. We modeled the first three SVD modes, comprising 73% of the global variation (Methods), which captured multiple biological processes (Table 2 and Table S3) and was suitable for the analysis of a relatively small sample size. Although there were substantially fewer samples than the phenotype data (92 versus 218 strains), the samples were selected for genetic diversity and the SVD modes provide more precise summary phenotypes because they are averaged over hundreds of genes. Therefore, the analysis was sufficiently powered to identify significant interactions and map a network model with five variant-to-variant influences (Figure S4 and Table S5). Three of these were identical in direction and sign to the ME/PR model (Figure 3), with two additional interactions that did not qualify as significant in the ME/PR model. The two independently-analyzed data sets thus uncover generally similar interaction networks with different details. We added additional SVD modes to the analysis on an exploratory basis and derived similar interactions but, as expected, found decreasing significance as signals were added. For example, the *fus3Δ* suppression of *msg5Δ* obtained in the ME/PR model (*p* = 0.006) was derived at similar interaction strength when taking three, four, or five SVD modes but with decreasing significance (*p* = 0.026, *p* = 0.030, and *p* = 0.076, respectively). This reduction in significance was due to adding additional signals without increasing population size.

## Discussion

Using only the genotype and phenotype information in our population, we identified many features of the mating pathway in haploid yeast. This serves as a strong validation of population-based analysis approach for mapping genetic interactions. Both the direct variant-to-phenotype influences and the suppressive variant-to-variant influences in our network (Figure 3) support previous findings that were based on targeted genetic perturbations. However, in some cases we did not find naively expected edges due to the averaging of direct influences over all models. While we found that the averaged influences were generally consistent, the averaging procedure occasionally resulted in insignificant mean values. For example, although we inferred a positive influence of *msg5Δ* on mating efficiency (Table S4) the averaged influence did not meet our significance criterion. The relative weakness of this effect was possibly compounded by its effect on cellular morphology during mating [33] that might be of lesser importance in the phenotypes we measured. Similarly, while *STE11-4* significantly increased both ME and PR when considered with *fus3Δ* (Figure 2D), its positive influence on ME was diluted and fell below significance when averaged over all pairs involving *STE11-4*. This was the result of averaging over the effects of all pair-wise regressions, and could lead to false negatives if real effects are averaged out. Nevertheless, the averaging procedure successfully identified effects missed in single-locus scans. We also note the variant-to-variant influences are uniquely determined and thus do not require averaging. However, resolving these genetic interactions requires that the phenotypes contain sufficient complementary information. Our network (Figure 3) represents the edges for which there is evidence in our particular data set rather than the map of all possible interactions. In this case we found that the complementary information is sufficient to recover an informative network of genetic interactions. Furthermore, we independently derived a network from a gene expression data that contained similar interactions (Figure S4), further validating our approach and results.

Because genetic interactions are always defined in the context of one or more specific phenotypes, the success of our technique crucially depends on the choice of phenotypes assayed. The quantitative accuracy and repeatability of molecular phenotypes such as gene expression are desirable for their large signal-to-noise potential. Indeed, we used microarray data to obtain a network of how interacting variants (Figure S4) combine to affect specific biological processes (Table 2 and Table S3). However, our method is also suitable to measurements at a higher level, such as quantitative cellular and physiological phenotypes, which often have greater sample sizes and are more directly related to a disease-related phenotype. Although our analysis technique involves recasting phenotypes in terms of maximum orthogonality, a crucial requirement is that the phenotypes manifest some degree of genetic overlap along with components of genetic independence. This reliance on partial pleiotropy suggests that the higher level phenotypes may be more powerful as they can involve a broader mix of partially overlapping biological processes. Indeed, the more complex biological basis of our colony-level mating efficiency assay provided the complementary information to our pheromone-specific molecular reporter necessary to successfully infer genetic interactions between alleles of mating genes.

Our method is flexible and extendable. In this study we inferred an interaction network with two quantitative phenotypes, which is the minimal but not limiting number. The analysis can be straightforwardly expanded for an arbitrary number of phenotypes (Methods, Eq. 5), and information gains can be expected for the portion of each added phenotype that is uncorrelated with existing phenotypes. However, in our gene expression analysis we found that the inclusion of additional orthogonal modes did not add novel information in terms of enriched GO processes. Furthermore, adding phenotypes without increasing the relatively small sample size weakened statistical significance. In other cases a larger sample size might resolve additional interactions and/or biological processes, but measuring genome-wide RNA abundances for a large number (e.g. hundreds) of samples remains expensive and cost constraints will often limit these measurements to a selected subset of the population based on genetic diversity or extreme phenotypic variability. In this study we followed this approach by using gene expression on a subset of samples to reveal the biology that underlies the cellular phenotypes, but our approach could have been readily applied to gene expression in the full population if resources were unlimited.

As with all statistical-based methods, our approach has analytical limitations. For more than two phenotypes a dimensional reduction occurs due to the fact that the approach is limited to pair-wise analyses and therefore a loss of fit accuracy compared to the combination of all pair-wise regressions for each phenotype independently. This is independent of the number of phenotypes (or SVD modes) fit and limits the amount of variance that can be modeled, while automatically maximizing information for each variant pair (Methods, Eq. 5). This limitation could be alleviated by performing analysis of three or more variants simultaneously [2], possibly by selectively analyzing interacting cliques in a pair-wise study, but extending beyond pair-wise analysis would likely be limited by the corresponding computational expense and statistical power requirements, especially in gene expression studies with limited sample size. The approach could also be minimally applied to interpret only locus pairs with statistically significant epistasis in one or more phenotypes; however this limited treatment may miss pair-wise interactions that become significant only when both phenotypes are jointly considered. In general, we expect the power requirements for our pair-wise approach to be equal to or less than that of standard pair-wise locus scans, with the potential power gain due to the simultaneous use of information in multiple phenotypes.

Because the analysis is defined in terms of variant effects it can theoretically address an arbitrary number of loci and can be used with multi-state models that consider multiple allele forms at each locus. In the multi-allelic case, each individual allele can be considered an independent network node. This would enable the inference of not only interactions for variants at different loci, but also intra-locus interactions to resolve allelic dominance [42]. Multiple alleles of the same gene could then be grouped according to similarity in interactions with all other variants independent of sequence similarity, allowing the inference of groups of alleles with similar gene activity such as loss or gain of function. In all cases, variant effects must be interpreted relative to the defined reference strain (Methods).

The calculations involved are individually fast and therefore the approach is scalable to many more than the five loci used here. For populations with very high genetic diversity that are genotyped or sequenced at high density, such as advanced intercross [43], [44], outcross [15], [16], or natural populations, substantial efficiency would likely be gained by pre-screening single-locus effects to prioritize potentially interacting loci. In sparsely-genotyped data augmented with imputed genotypes (e.g. Haley-Knott regression approach [45]), our method can be used to infer interactions between variants at quantitative trait loci. Finally, the approach could also be adapted to large-scale genetic interaction screens in which multiple phenotypes are measured.

Casting genetic interactions in terms of quantitative, directional variant-to-variant influences provides specific hypotheses for genetic interactions. Determining the direction of interaction between gene variants constrains possible interpretations and facilitates more specific designs for experimental validation. For example, it enables focused integration with complementary molecular data and thereby increases predictive power [2]. Furthermore, models that quantify changes in the activity of gene variants can enable predictions of the phenotypic effects of alternative alleles of the same gene as well as small molecules and biologics affecting a gene or gene product, when the alleles or agents can be characterized as a loss or gain of function [46]. Our approach therefore provides a method to reanalyze existing genetic data to translate statistical epistasis into precise and testable hypotheses.

## Methods

### Yeast Strains

We constructed two parental strains of opposite mating types that were mating-competent and together harbored all mutations (Text S1). We mated, sporulated, and selected one *MATa* progeny per tetrad to obtain a population of 218 strains with independent assortment of the five genetic variants (Figure S1). The parental strains were derived from BY4741/2 haploids with an enhanced green fluorescent protein (GFP) gene under the control of a minimal promoter with tandem pheromone response elements from the *PRM1* promoter [47] inserted at the *HIS3* locus [33]. We genotyped the deletion strains with drug-resistant markers and verified genotypes for the strains for which we collected microarray data (see below). Eighteen genotypes at the *MSG5* locus were corrected due to the relatively error-prone DsdA insertion marker (Text S1). *STE11-4* was genotyped using Invitrogen Quant-iT Picogreen dsDNA quantitation and Sequenom hME genotyping, all following manufacturer protocols. Allele frequencies were balanced at approximately 50% across the population for all mutations except the *STE11-4* allele, which was underrepresented by 10%. This was probably due to its associated growth defect. Our population contained one or more progeny for 29 of the 32 possible genotypes, with missing genotypes due to synthetic growth defects or random chance.

### Phenotyping

Mating efficiency (ME) was measured by culturing each of our *MATa* leucine, methionine, and uracil auxotroph strains with an excess of *MATα* leucine, lysine, and uracil auxotroph [48]. We combined and gently pelleted strains grown in rich media and incubated without agitation for 5 hr at 30 C in order to allow mating. Cultures were washed and plated on synthetic dextrose media supplemented with (i) leucine and uracil to select for diploids, and (ii) leucine, uracil, and methionine to permit growth of both diploids and the *MATa* haploid strain of interest as a control. A range of dilutions was used to encompass the large dynamic range in growth and mating proficiency. From initial cultures at OD_600_ = 1, 25 µL serial dilutions of 1∶10, 1∶100, and 1∶1000 were plated on diploid-selective media and dilutions of 1∶250, 1∶1000, and 1∶2500 on control media using a Genetix QP Expression benchtop colony picking system. Colony growth was quantified by digital images and ImageJ software [49], and data were selected from the dilutions that exhibited moderate growth with clearly identifiable colonies (Figure S2). ME was scored as a ratio of diploid to control pixel counts with appropriate normalization for differences in strain dilution. Average values over two replicates were log-transformed. Six strains with zero average mating efficiency were assigned values at the extreme low end of the distribution. After processing the data formed a nearly-symmetric, unimodal distribution.

Pheromone response (PR) was quantified as the activity of the canonical mitogen-activated protein kinase (MAPK) pathway measured by the signal of an enhanced green fluorescent reporter (GFP) driven by a pheromone response element (PRE) binding site. We collected samples after 3 hours incubation at 30 C in synthetic complete media with 1 µM α-factor. Prior to flow cytometry analysis, collected cells were forced three times through a 25 1/2-gauge needle to separate cells in chains or break up cell aggregates [50]. Successful cell separation was verified microscopically. GFP fluorescence was measured using a BD Biosciences FACSCalibur flow cytometer. Log intensities were measured for two replicates of each strain and averaged to yield PR values. The log-transformed intensities formed a nearly-symmetric distribution with a single broad peak in the center. Both phenotypes were mean-centered and standard-deviation normalized to remove arbitrary scale differences in the analysis.

### Genetic Interaction Analysis

We developed a novel data analysis technique to interpret genetic interactions between variants in the population as quantitative influences (Figure 1). Although our genotyping was limited to five variant loci, the method was also designed for genotypes defined by SNP or microsatellite markers, full sequence information, imputed genotype probabilities, or other standard methods.

To fully exploit complementary information in multiple phenotypes we analyzed linear combinations that maximized orthogonality. These were defined by singular value decomposition (SVD) of a matrix of mean-centered and standard-deviation-normalized data, 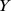, with a row for each of the 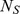 samples and a column for each of the 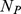 phenotypes:
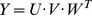
(1)Assuming more samples than phenotypes, the 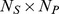 matrix 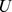 contains the left singular vectors in columns, the diagonal 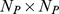 matrix 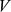 contains the singular values, and the 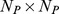 matrix 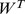 contains the right singular vectors in rows. Each left singular vector is a composite phenotype for all samples. For our data, the two columns of 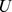 were the normalized sum and difference of our original phenotypes ME and PR. We refer to these uncorrelated composite phenotypes as eigentraits ET1 and ET2, respectively. Since there is no dimensional reduction our original phenotypes can be reconstructed from our analysis on eigentraits without loss in fit quality.

We first performed linear regression for each of the five perturbed genes in isolation to identify strong-effect variants that will be treated as covariates in subsequent pair-wise regressions. For each locus we consider the model:
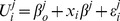
(2)The index 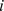 is from 1 to 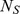 and 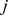 is from 1 to 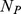. The variable 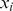 is the probability of the gene variant at the locus in the strain 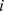, 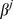 is the effect on the phenotype 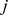, and 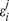 is the residual error. For our data all 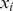 were binary, although the approach can be generalized to multiple alleles at the same locus with arbitrary probabilities. In that case, each allele has a unique effect and the locus might contain multiple strong-effect variants. We note that this procedure defines a single reference allele for each locus (here the wild-type) with the consequence that the resulting network is defined relative to this baseline allele and will not necessarily be invariant for a change in reference. For each locus, we defined strong-effect variants as those with a significant effect (see below). We conditioned each subsequent allele-pair scan by including strong-effect variants as covariates for the associated phenotype. Performing scans on eigentraits optimizes the choice in covariates, but if no strong effect variants are found then analyzing the original phenotypes will lead to identical results.

We next modeled every possible variant pair with main effects and an interaction term, taking the strong-effect variants as covariates. For two alleles labeled 1 and 2 we have:
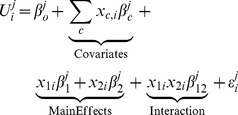
(3)The variables are defined as in Eq. 2 with the additional interaction coefficient 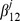 and sum over strong-effect variants as covariates (excluding alleles 1 and 2). Statistical epistasis is the occurrence of a significant interaction coefficient in this pair-wise regression. Each variant pair has an independent interaction term for each phenotype.

To derive a model in terms of variant-to-variant influences, we first reparametrized the set of interaction coefficients in terms of two new variables, 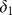 and 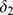. The value of 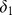 represents the inferred change in activity of the first perturbed gene when the second perturbation is present. Each 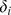 is posited to be independent of phenotype. This represents genetic interactions by quantifying the degree to which the two variants modify one another's influence on the downstream phenotypes. For example, one variant suppressing another will result in a negative 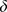 coefficient, indicating the phenotype-relevant activity of the suppressed variant is reduced. The effects of this change in activity on each phenotype were weighted by the main effect coefficient of the perturbation on that phenotype. For each phenotype we defined:
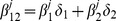
(4)This recast the 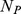 interaction coefficients 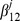 in terms of the modified activity parameters 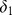 and 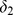 that are independent of 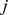. This reparametrization of interaction coefficients is the fundamental idea of our approach. The activity variables were computed by matrix inversion:
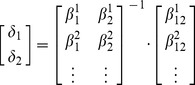
(5)For two phenotypes, such as ME and PR, the variables 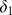 and 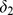 were an exact reparametrization of the interaction coefficients. For more than two phenotypes, such as our expression modeling which involved three, the inverted main-effect matrix can be pseudoinverted for a least-squares fit.

We next translated the activity variables into two influence coefficients that quantify the variant to-variant influences. This quantifies the influence that is necessary for one variant to modify the activity of the other, as modeled with the 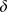 coefficients. The result is a map of how each variant influences the others, with negative influences correspond to suppression and positive influences correspond to enhancement. We rewrote the change in activity of variant 1 as an interaction coefficient (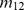) times the activity of variant 2, and vice-versa:
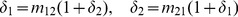
(6)The solutions for the influence coefficients are:
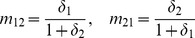
(7)By substituting the solution of Eq. 5 into Eq. 7 we obtained the influence coefficients 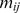 as a function of the regression parameters. This defined our model in terms of variant-to-variant influence coefficients (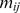) and a matrix of main-effect, variant-to-eigentrait coefficients (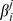). Without loss of information, we multiplied the eigentrait coefficient matrix by the singular value matrices 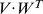 to obtain variant-to-phenotype coefficients for ME and PR (Text S1).

We assessed the significance of the influence coefficients, 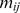, and variant-to-phenotype coefficients using standard error analysis methods on the regression parameters [51]. For example, the variance of 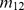 is estimated by differentiating with respect to all model parameters:
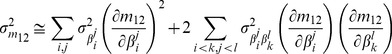
(8)The indices 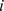 and 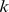 run over regression parameters and 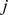 and 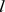 run from 1 to 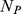. The first sum is over individual parameters and the second double sum is the cross terms. Variances and covariances were estimated from the least-squares regression using standard methods [51]. Error analysis was of particular importance because matrix inversions can often lead to large values with larger standard errors and overall insignificant results.

After computing models for all variant pairs, we obtained two variant-to- variant influence coefficients between each variant pair (one in each direction) and variant-to-phenotype coefficients for every model that included a given variant. We averaged the latter values to estimate overall variant-to-phenotype coefficients and computed the corresponding root-mean-square error (Eq. 8). To determine significance we used effect size divided by estimated standard error as our test statistic because it could be computed for both regression coefficients and variables computed from them (e.g. 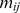). We first determined significance for single-locus scans. We performed 10,000 permutations of the genotype data and fit an extreme value distribution (EVD) for the maximum test statistic from each permutation. This accounts for multiple tests and empirically estimates the likelihood of chance association. We determined that a test statistic of 3.55 or greater corresponds to *p*<0.01. We repeated the procedure for pair-wise scans, permuting the genotypes of the two markers being tested in tandem. This procedure retains any linkage between the two markers and randomizes only the marginal effects after conditioning on covariates. We collected test statistics for 100,000 permutations to obtain null distributions for variant-to-phenotype and variant-to-variant influence coefficients. We computed an empirical *p*-value for each coefficient. Family-wise error rate was controlled by adjusting p-values using a step-down procedure [52]. Step-down EVDs were not used because the empirical distributions had slightly greater support at higher values than fitted EVDs and thus the EVDs would artificially inflate significance. A significance cutoff of adjusted *p*<0.05 was used in our network (Figure 3) and all estimated p-values are reported in Table S4.

### Gene Expression Analysis

We used gene expression analysis to identify the different biological processes that underlie the ME and PR phenotypes. We collected 96 samples representing 28 unique genotypes in our population, with the majority of genotypes represented by three independently-derived segregants. Samples were collected after 3 hours of agitated incubation in synthetic complete media with 1 µM α-factor. Cells were prepared with Qiagen RNeasy Mini kits and target labeling was performed using Agilent One-Color Microarray-Based Gene Expression Analysis kit and protocols. Samples were hybridized to Agilent (V2) Yeast Gene Expression Microarrays following manufacturer's protocols. Signals from duplicated probes were averaged. For most genotypes without three independent segregants in our population we obtained biological replicates; however one of the genotypes is represented by a single sample and four were represented by two samples. Expression intensities for each gene were expressed as log2 ratios relative to mean expression of the unperturbed strain (wild-type) under the same experimental conditions.

We used SVD to rearrange the data into a series of global expression patterns, or modes, and sets of genes that show those patterns [38]. SVD was performed on the data matrix of all 6208 genes plus two phenotypes by the 92 perturbed strains, mathematically separating the data matrix into a set of ‘modes’ defined by quantitative patterns within the data. Each mode is manifest in the data as a global expression pattern that contributes to the expression of each gene to a degree varying from negligible to predominant. The expression of each gene can be expressed as a linear combination of these global patterns, each multiplied by the entries of a weight vector unique to that gene. We followed the procedures in a previous publication [53] to identify gene sets associated with each mode and to query those sets for enriched Gene Ontology annotations and transcription factor targets. Briefly, for each expression mode we identified genes with mode weights of two or more standard deviations from the mean. For each mode, significant genes with positive weights were assigned membership in one set and those with negative weights in another, composing two gene sets. These gene sets (Table 2 and Table S3) were queried for enrichment of GO [54] biological process annotations and transcription factor targets [55]–[57] using Fischer's exact test (Table 2 and Table S3). Since GO annotations have a large degree of interdependence, we performed permutation tests on gene names and estimated a false discovery rate of 0.6% for our *p*-value cutoff of *p*<10^−4^, suggesting less than one in one hundred of our enrichments are false positives. Transcription factor targets were Bonferroni corrected for the 119 assessed factors.

### Gene Expression Data

Gene expression data have been deposited in the Gene Expression Omnibus, accession GSE34787.

## Supporting Information

Figure S1 Yeast intercross design. Two parental strains of indicated genotypes were mated and the resulting diploid was sporulated to produce 218 haploid strains, each selected to be *MATa* but otherwise harboring a random assortment of mutations.
(PNG)

Figure S2 Mating efficiency assay. Four representative strains ranging from low (48A, left) to high (51B, right) mating efficiency. Diploid rows show colonies grown from successful mating. Diploid+haploid row shows combination growth of the named strain mated diploids, which serves as a growth control. Values are ratio of pixel counts for the most readily quantified dilution (multiple, well-separated colonies) to the growth control (bottom row).
(PNG)

Figure S3 Gene expression for the first three SVD modes. Strains are separated by genotype.
(PNG)

Figure S4 Combined genetic influence network for SVD expression modes, mapping positive (green) and negative (red) influences between mutations and on the expression phenotypes. Edge width represents interaction strength.
(PNG)

Table S1 Strain genotypes and phenotypes.
(XLSX)

Table S2 SVD gene sets.
(XLSX)

Table S3 SVD gene set GO biological process annotation and transcription factor enrichments for gene expression sets.
(XLSX)

Table S4 Results for all computed influence parameters for ME and PR phenotypes.
(XLSX)

Table S5 Results for all computed influence parameters for gene expression modes.
(XLSX)

Text S1 Supplemental text containing details of strain constructions and methods.
(DOCX)
