# Supplementary figures and images for: Use of Pleiotropy to Model Genetic Interactions in a Population
Source: PLoS Genet. 2012 Oct 11;8(10):e1003010. doi: 10.1371/journal.pgen.1003010 (PMC3469415; doi:10.1371/journal.pgen.1003010)

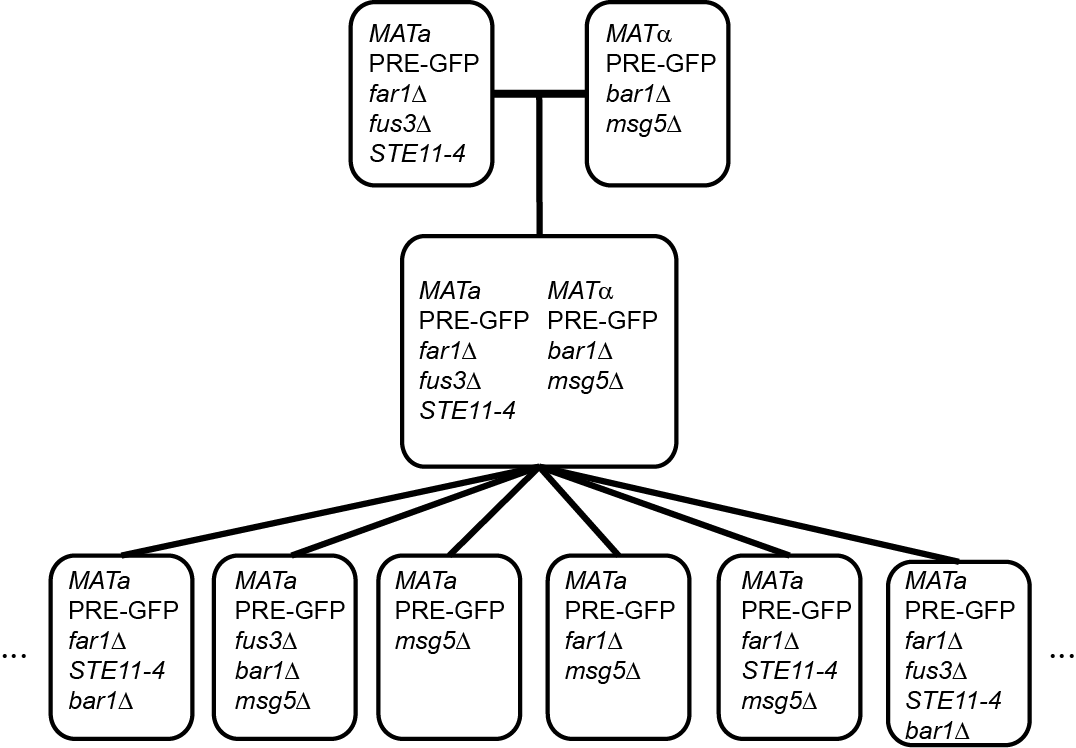

Supplement: Figure S1 — Yeast intercross design. Two parental strains of indicated genotypes were mated and the resulting diploid was sporulated to produce 218 haploid strains, each selected to be MATa but otherwise harboring a random assortment of mutations. (PNG) [file pgen.1003010.s001.png]

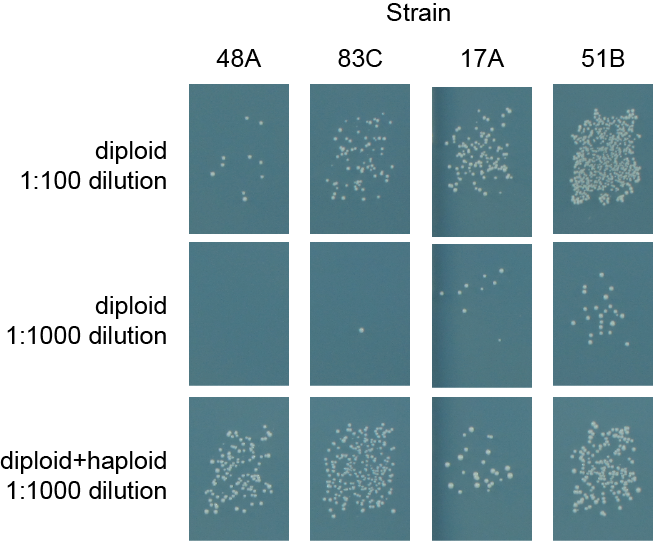

Supplement: Figure S2 — Mating efficiency assay. Four representative strains ranging from low (48A, left) to high (51B, right) mating efficiency. Diploid rows show colonies grown from successful mating. Diploid+haploid row shows combination growth of the named strain mated diploids, which serves as a growth control. Values are ratio of pixel counts for the most readily quantified dilution (multiple, well-separated colonies) to the growth control (bottom row). (PNG) [file pgen.1003010.s002.png]

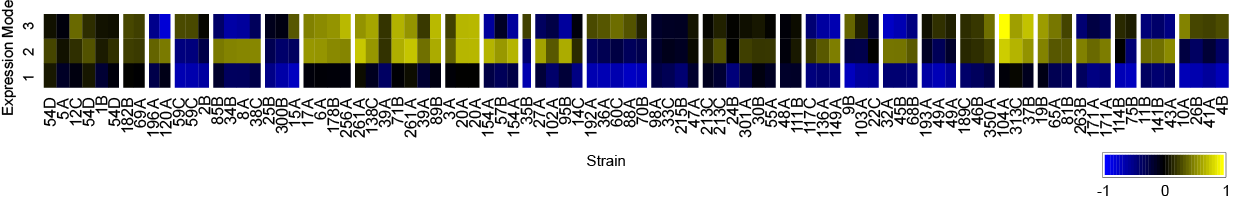

Supplement: Figure S3 — Gene expression for the first three SVD modes. Strains are separated by genotype. (PNG) [file pgen.1003010.s003.png]

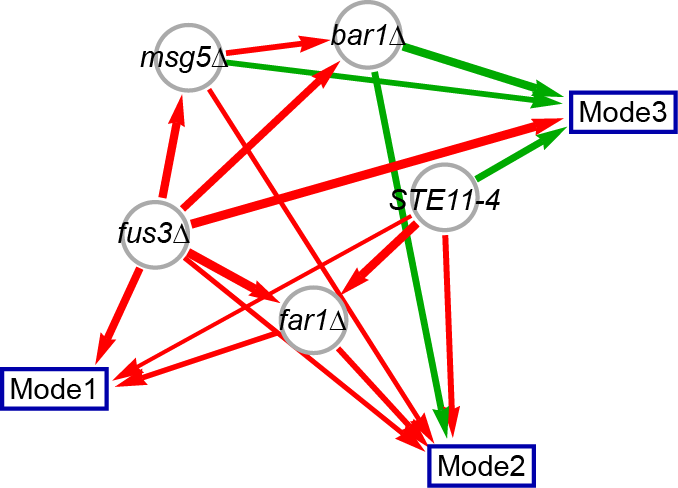

Supplement: Figure S4 — Combined genetic influence network for SVD expression modes, mapping positive (green) and negative (red) influences between mutations and on the expression phenotypes. Edge width represents interaction strength. (PNG) [file pgen.1003010.s004.png]
