# Supplementary material for: Use of Pleiotropy to Model Genetic Interactions in a Population
Source: PLoS Genet. 2012 Oct 11;8(10):e1003010. doi: 10.1371/journal.pgen.1003010 (PMC3469415; doi:10.1371/journal.pgen.1003010)
Supplement: Text S1 — Supplemental text containing details of strain constructions and methods. (DOCX) [file pgen.1003010.s010.docx]

**Use of Pleiotropy to Model Genetic Interactions in a Population**

Gregory W Carter, Michelle Hays, Amir Sherman, Timothy Galitski

**SUPPORTING TEXT**

**Strain Construction**

All strains in the yeast population were derivatives of BY4741/2 haploids with a PRE-GFP insertion at the *HIS3* locus [[1](#_ENREF_1)], genotype *MATa leu2Δ0 met15Δ0 ura3Δ0 his3::HIS3::PRE-GFP.* Gene deletions were made using a PCR-based strategy in which the KanMX4 “barcode” deletion allele [[2](#_ENREF_2)], the NatMX4 allele [[3](#_ENREF_3)], and DsdAMX4 [[4](#_ENREF_4)] allele were amplified, transformed into our progenitor strain, and verified by PCR and tetrad dissection. Standard methods were used for all transformations [[5](#_ENREF_5)]and crosses to construct haploid mutant derivatives. Plasmid pMKV001 containing the DsdA allele was kindly provided by the Dudley Lab at Institute for Systems Biology.

The *bar1::HphMX4* gene deletion was obtained from a previous study [[1](#_ENREF_1)]. The *fus3::NatMX4* gene deletion was constructed using primers Fus3Nat_F (5’ - AG GCA GAG AAA AAG AAA GGA AAA TAA TAT GCC AAA GAG GCA TAG GCC ACT AGT GGA TCT G – 3’) and Fus3Nat_R (5’ - T GTT CTT CGG GTT GAT ATT TTA ATG ATA ATG ATG GCT CAG CTG AAG CTT CGT ACG C - 3’). The *far1::KanMX4* gene deletion was constructed using primers Far1_KanMX4_F (5’ – AAA AGC AAA AGC CTC GAA ATA CGG GCC TCG ATT CCC GAA CTA CTA GCA TAG GCC ACT AGT GGA TCT G – 3’) and Far1_KanMX4_R (5’ – ATC CAC TGG AAA GCT TCG TGG GCG TAA GAA GGC AAT CTA TTA ATG CAG CTG AAG CTT CGT ACG C – 3’). The *msg5::DsdA* gene deletion was constructed using primers Msg5_dsdA_F (5’ – AAA CAT TCT TTT TTT TTT CTT CAT TTA AGT ATA AGA CAA CAA TAG TAG CGG AAG CGA TAG TAA GCA TAG GCC ACT AGT GGA TCT G – 3’) and Msg5_dsdA_R (5’ – GCT AAG TAC ATT CTA TTT AGA AGC GTA GGG AGG CGG TCT GCC TGC AGA TCT GAT AGT CAT AAA CAG CTG AAG CTT CGT ACG C – 3’).

The *STE11-4* allele, a C→T substitution at gene position 1787 (ChrXII:851652), was constructed with two-step gene replacement (Prinz, Shelby, and Galitski, unpublished). A URA3 gene was amplified from plasmid pRS306 [[6](#_ENREF_6)] and inserted into the STE11 gene using primers ste11_1787::URA_F (5’ – GCA AAT ATT TTG ATT GAT ATC AAA GGT TGC GTA AAA ATT ATA ACT ATG CGG CAT CAG AGC – 3’) and ste11_1787::URA_R (5’ – TTT ATT CAA AGG TGA TAA TTT TTT TGA AAT ACC AAA ATC ACC TGA TGC GGT ATT TTC TCC – 3’). The *URA3* was ejected using primers ste11T596I_F (5’ – CAG AGA TAT CAA GGG TGC AAA TAT TTT GAT TGA TAT CAA AGG TTG CGT AAA AAT TAT TGA TTT TG – 3’) and ste11T596I_R (5’ – CTC TCT TAT TTT GTT TTT TAT TCA AAG GTG ATA ATT TTT TTG AAA TAC CAA AA TCA ATA ATT TTT – 3’). *STE11-4* strains were selected on 5-FOA media.

Mutations were integrated sequentially to produce the parental genotypes *MATa fus3::NatMX4 far1::KanMX4 STE11-4 leu2Δ0 met15Δ0 ura3Δ0 his3::HIS3::PRE-GFP* and *MATα bar1::HphMX4 msg5::DsdAMX4 leu2Δ0 met15Δ0 ura3Δ0 his3::HIS3::PRE-GFP.* These strains were mated, sporulated, and tetrads dissected using standard methods. All tetrads were scored for mating type and drug resistances and a single *MATa* progeny from each tetrad was selected for *STE11* genotyping by Sequenom hME.

**Matrix Formulation of Reparametrizaton**

The model can be expressed in matrix form, with $N$ samples over $M$ phenotypes and $C$ covariates:

$$\begin{matrix} \left[\begin{matrix} U_{1}^{1} & U_{1}^{2} & \cdots& U_{1}^{M} \\ U_{2}^{1} & U_{2}^{2} & \cdots& U_{2}^{M} \\ \vdots& \vdots& \ddots& \vdots\\ U_{N}^{1} & U_{N}^{2} & \cdots& U_{N}^{M} \end{matrix}\right]=\left[\begin{matrix} 1 & x_{c1,1} & \cdots& x_{1,1} & x_{2,1} & x_{1,1}x_{2,1} \\ 1 & x_{c1,2} & \cdots& x_{1,2} & x_{2,N} & x_{1,2}x_{2,2} \\ \vdots& \vdots& \ddots& \vdots& \vdots& \vdots\\ 1 & x_{c1,N} & \cdots& x_{1,N} & x_{2,N} & x_{1N1}x_{2,N} \end{matrix}\right]\cdot\left[\begin{matrix} \beta_{0}^{1} & \beta_{0}^{2} & \cdots& \beta_{0}^{M} \\ \beta_{c1}^{1} & \beta_{c1}^{2} & \cdots& \beta_{c1}^{M} \\ \vdots& \vdots& \ddots& \vdots\\ \beta_{1}^{1} & \beta_{1}^{2} & \cdots& \beta_{1}^{M} \\ \beta_{2}^{1} & \beta_{2}^{2} & \cdots& \beta_{2}^{M} \\ \beta_{12}^{1} & \beta_{12}^{2} & \cdots& \beta_{12}^{M} \end{matrix}\right] \\ =\left[\begin{matrix} 1 & x_{c1,1} & \cdots& x_{1,1} & x_{2,1} & x_{1,1}x_{2,1} \\ 1 & x_{c1,2} & \cdots& x_{1,2} & x_{2,N} & x_{1,2}x_{2,2} \\ \vdots& \vdots& \ddots& \vdots& \vdots& \vdots\\ 1 & x_{c1,N} & \cdots& x_{1,N} & x_{2,N} & x_{1N1}x_{2,N} \end{matrix}\right]\cdot\left[\begin{matrix} 1 & 0 & \cdots& 0 & 0 \\ 0 & 1 & \cdots& 0 & 0 \\ \vdots& \vdots& \ddots& \vdots& \vdots\\ 0 & 0 & \cdots& 1 & 0 \\ 0 & 0 & \cdots& 0 & 1 \\ 0 & 0 & \cdots& \delta_{1} & \delta_{2} \end{matrix}\right] \\ \cdot\left[\begin{matrix} \beta_{0}^{1} & \beta_{0}^{2} & \cdots& \beta_{0}^{M} \\ \beta_{c1}^{1} & \beta_{c1}^{2} & \cdots& \beta_{c1}^{M} \\ \vdots& \vdots& \ddots& \vdots\\ \beta_{1}^{1} & \beta_{1}^{2} & \cdots& \beta_{1}^{M} \\ \beta_{2}^{1} & \beta_{2}^{2} & \cdots& \beta_{2}^{M} \end{matrix}\right] \end{matrix}$$

If we write this as $U=X\cdot\delta\cdot\beta$, it follows that the original phenotype matrix is

$$Y=X\cdot\delta\cdot\beta\cdot V\cdot W^{T}$$

We define the direct influences as $\beta'=\beta\cdot V\cdot W^{T}$ to recompose the SVD and have $Y=X\cdot\delta\cdot\beta'$. This is our final result, in which the phenotype $Y$ is modeled as a genotype design $X$ (the design matrix of the pair-wise regression), the interaction matrix $\delta$ that contains the interactions that can be expressed in terms of variant-to-variant influences $m_{12}$ and $m_{21}$, and the coefficient matrix $\beta'$ that contains the direct effects on the phenotype from the variant pair and covariates.

**SUPPORTING REFERENCES**

1. Taylor RJ, Falconnet D, Niemisto A, Ramsey SA, Prinz S, et al. (2009) Dynamic analysis of MAPK signaling using a high-throughput microfluidic single-cell imaging platform. Proceedings of the National Academy of Sciences of the United States of America 106: 3758-3763.

2. Winzeler EA, Shoemaker DD, Astromoff A, Liang H, Anderson K, et al. (1999) Functional characterization of the S. cerevisiae genome by gene deletion and parallel analysis. Science 285: 901-906.

3. Goldstein AL, McCusker JH (1999) Three new dominant drug resistance cassettes for gene disruption in Saccharomyces cerevisiae. Yeast 15: 1541-1553.

4. Vorachek-Warren MK, McCusker JH (2004) DsdA (D-serine deaminase): a new heterologous MX cassette for gene disruption and selection in Saccharomyces cerevisiae. Yeast 21: 163-171.

5. Guthrie C FG (1991) Guide to yeast genetics and molecular biology. New York: Academic Press.

6. Sikorski RS, Hieter P (1989) A system of shuttle vectors and yeast host strains designed for efficient manipulation of DNA in Saccharomyces cerevisiae. Genetics 122: 19-27.
